# NASH-related Hepatocellular carcinoma: current therapeutic approaches and the emerging role of sodium homeostasis as a novel targeting strategy

**DOI:** 10.3389/fphar.2025.1671001

**Published:** 2025-09-03

**Authors:** Abdulsalam Ashkar, Simone Fiorilla, Francesco Tasso, Rita Carini

**Affiliations:** Department of Health Science Università del Piemonte Orientale, Novara, Italy

**Keywords:** hallmarks of cancer, Hepatocellular carcinoma, Monensin, non-alcoholic fatty liver disease, cancer targeting treatment, sodium homeostasis, Warburg effect

## Abstract

Non-Alcoholic Steatohepatitis (NASH) rates are progressively accelerating due to lifestyle changes, which contribute to increased Hepatocellular carcinoma (HCC) incidence. HCC accounts for 90% of liver cancer cases, which ranks as the sixth prevalent, and the third leading cause of cancer-related deaths globally. NASH-HCC outweighs the decline in viral hepatitis-HCC, leaving a gap in the available therapies. The limited overall survival in the current treatments invokes the necessity of exploring novel therapeutic strategies to improve the poor prognosis of HCC. The pH gradient is a hallmark of cancer and is associated with increased intracellular sodium. Elevating this accumulation of intracellular sodium with sodium ionophores, such as Monensin, leads to selective death of murine HCC cancer cells without affecting the functionality of vital organs and proliferating activity of normal and transformed tissues. This study synthesizes the status of HCC risk and management, its molecular landscape, and sheds light on exploiting the elevated accumulation of intracellular sodium as a novel therapeutic strategy against HCC.

## 1 Introduction

Liver cancer is the sixth most frequently diagnosed cancer and the third leading cause of death in cancer-related mortalities worldwide. In 2025, about 1 million new incidents are predicted to occur compared with 840,000 in 2018 whereas fatalities are foreseen to rise by more than 50% by 2040 (Rumgay et al., 2022). HCC represents roughly 90% of liver malignancies, with incidence rates increasing with age (Llovet et al., 2021).

HCC global epidemiology is shifting, with steady increases in the projected incidence in the upcoming 30 years. Hepatitis B virus (HBV) infection was considered the most common risk factor for HCC. Recent advances in HBV vaccination, treatments, and preventive measures for HBV and the Hepatitis C virus (HCV) have reduced the global incidence. For instance, Direct-acting antiviral (DAA) reduced the risk of HCC by 50%–80%. While viral hepatitis caused by HBV and HCV is dipping, NASH-related Hepatocellular Carcinoma (HCC) prevalence is widely increasing, in a pattern that overweighs the decline in viral hepatitis-related HCC, leading to a net increase in overall incidence (Koshy, 2025). These global trends are mostly attributed to increasing obesity, diabetes, and NASH prevalence (Llovet et al., 2021). However, up to 25% of HCC cases occur in patients without a history of cirrhosis or its associated risk factors (Medscape, 2024).

## 2 NAFLD development into HCC

**FIGURE 1:**
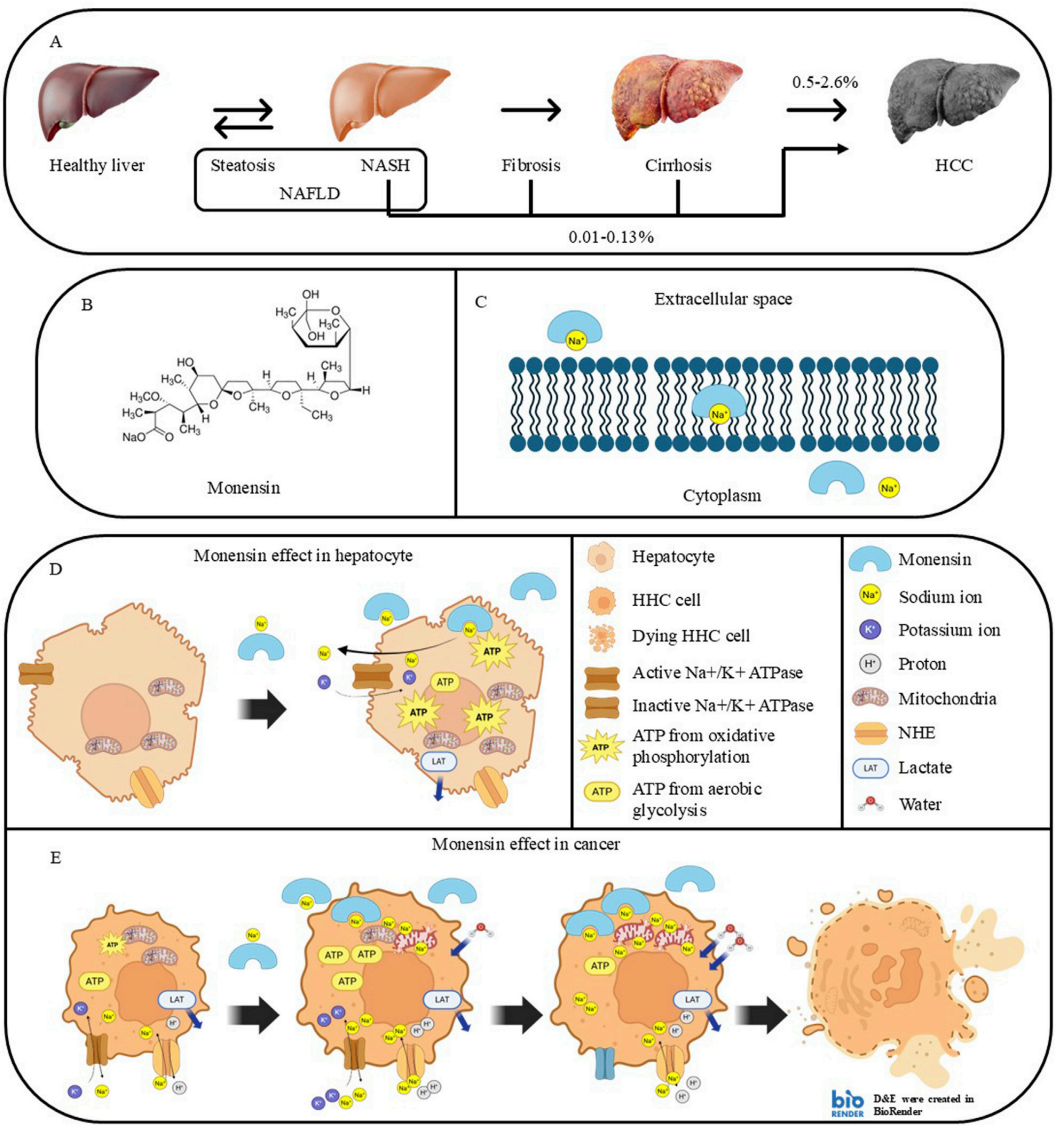
NASH progression to HCC and proposed mechanisms involved in the cancer-selective Monensin cytotoxicity. **(A)** Stages of liver pathologies during developing NASH-related HCC. **(B)** Chemical structure of Monensin (C36H61NaO11). **(C)** Mechanism of action of Monensin. Monensin is a lipophilic compound that can form a complex with sodium, facilitating its transport through the cell membrane. **(D,E)** Proposed mechanisms involved in the cancer selective Monensin cytotoxicity: **(D)** In hepatocytes, Monensin leads to increased intracellular sodium, which is resolved by sodium-regulating proteins, mainly Na+/K + ATPase, using ATP produced by oxidative phosphorylation. **(E)** In HCC cells, Basal high intracellular sodium, related to excessive production of lactate from aerobic glycolysis, which requires exchange of lactate and protons with sodium. Monensin further elevates the intracellular sodium accumulation that leads to sodium-dependent mitochondrial dysfunction, continuous ATP loss, and water retention. This leads to an irreversible energetic and osmotic stress with lysis and death of the HCC cells.

Non-alcoholic fatty liver disease (NAFLD) is the most globally prevalent chronic liver disease, which affects 25% of the global population. Hepatic steatosis evolves into nonalcoholic steatohepatitis (NASH), which is the fastest-growing leading cause of HCC, through a series of fibrosis and cirrhosis if left untreated (Figure 1A) (Zhang et al., 2023). 0.5%–2.6% and 0.01%–0.13% of NASH-related cirrhosis and non-cirrhotic NAFLD evolve into HCC annually, respectively. Even though the incidence of developing HCC from NAFLD is lower than developing it from viral hepatitis, the prevalence of viral hepatitis is much less than the prevalence of NASH (Huang et al., 2021). Steatosis is a reversible process led by the accumulation of lipids in the liver. The accumulated lipids can lead to chronic inflammation, reactive oxygen species (ROS) generation, and lipid peroxidation. The chronic inflammation and persistent injury activate hepatic stellate cells, leading to extracellular matrix deposition and fibrosis. DNA damage from oxidative stress and chronic inflammation induces mutations in cancer driver genes, leading to malignant transformation (Figure 1A) (Wegermann et al., 2021).

## 3 Diagnostics and clinical manifestations

Typical cases of HCC progress without visible symptoms, complicating the early detection. Cirrhosis can usually be accompanied by liver reserve reduction, resulting in decompensation symptoms like jaundice, encephalopathy, ascites, or variceal bleeding, often associated with portal invasion. While non-cirrhotic patients, more common in high-incidence areas, exhibit serious symptoms including cachexia, abdominal pain, hepatomegaly, or jaundice due to unrestricted tumor growth. Tumor rupture is rare, but it results in hypotension, acute pain, and peritoneal irritation. The most common metastasis locations associated with HCC include abdominal organs, lungs, and bones. HCC can sometimes cause paraneoplastic effects such as hypoglycemia, diarrhea, or cutaneous signs. Porphyria cutanea tarda, linked to hepatitis C, is associated with an increased risk of HCC (Bialecki and Di Bisceglie, 2005).

High plasma levels of Alpha-fetoprotein (AFP) can indicate malignant growth from the same endodermal lining as the hepatic diverticulum, such as the stomach and pancreas. AFP has almost 100% specificity, but low sensitivity. Half the patients may not have diagnostic levels of AFP (400–500 ng/mL), with 30% of patients showing normal serum levels of AFP (below 10 ng/dL) when diagnosis is established (Ayuso et al., 2018). AFP can be replaced or combined with ultrasound imaging, increasing the Positive Predictive Value to 94%, it can also detect masses smaller than 3 cm (Bialecki and Di Bisceglie, 2005). Ultrasonography (US) is recommended every 6 months alone or with AFP to screen cirrhotic and high-risk patients. magnetic resonance imaging (MRI), multiphase computed tomography (CT), or a biopsy can be indicated to confirm the ultrasonography results.

Liver function tests (LFTs), complete blood cell count (CBC), electrolyte levels, and coagulation tests are indicated for recently diagnosed patients for severity assessment. Diagnosis can be established based on non-invasive imaging alone, regardless of the biopsy; it is also necessary to guide the biopsy if indicated (medscape, 2024). Key histological characteristics of HCC identified by the International Consensus Group for Hepatocellular Neoplasia include widespread fatty changes, intra-tumoral portal tracts, stromal invasion, pseudo-glandular architecture, elevated cell density, and unpaired arteries (RONCALLI, 2009). Barcelona Clinic Liver Cancer (BCLC) staging system and the National Comprehensive Cancer Network (NCCN) guidelines are generally followed to stage and manage the disease (Reig et al., 2022).

## 4 Current therapies

### 4.1 Surgical and locoregional approaches

The current gold-standard treatment for HCC in its early stages is Orthotopic liver transplantation (OLT), yet less than 10% of patients are eligible. Locoregional therapies are therefore crucial, including Transarterial embolisation (TAE) and chemoembolisation (TACE) that obstruct arterial supply, and deliver local chemotherapy with TACE, which is effective for tumors 3–5 cm. Whereas Radioembolisation (TARE) delivers internal radiation via hepatic arteries. Ablative techniques such as radiofrequency ablation (RFA), cryoablation, microwave ablation, and newer methods like IRE, HIFU, and LITT, can be selected based on tumor size, location, and liver function (Makary et al., 2020).

### 4.2 Targeted therapies

Targeted therapy has provided a substantial advancement in HCC management, namely, anti-angiogenic agents, such as Vascular Endothelial Growth Factor (VEGF) signaling targeted agents. Bevacizumab is a humanized monoclonal antibody that binds and inhibits VEGF-A, which has demonstrated a better outcome in combination with immunotherapies. Ramucirumab is a fully human IgG1 that targets VEGFR-2, which in Phase III REACH-2 trial achieved a higher response and disease control rates (5% vs. 1%, 60% vs. 39%, respectively), and improved overall survival (OS) (8.5 vs. 7.3 months; HR 0.71; 95% CI 0.531–0.949) against placebo in HCC patients with post-sorafenib progression (Zhu et al., 2019). The multi-kinase inhibitor (TKI) sorafenib has shown superior efficacy in HCV-related HCC (14 vs. 7.4 months), and its survival benefits were confirmed in SHARP and a phase III Asia-Pacific region trials (10.7 vs. 7.9 months and 6.5 vs. 4.2 months, respectively) (Llovet et al., 2008; Cheng et al., 2009). While Orantinib did not improve OS in phase-III ORIENTAL (Taiho Pharmaceutical Co., Ltd, 2019). Apatinib (targets VEGF-2) and lenvatinib (multi-TKI) have shown outcomes like sorafenib. Other TKIs like regorafenib and cabozantinib can be considered in second-line treatment following post-sorafenib progression, with favorable pharmacokinetic properties associated with donafenib (Niu et al., 2021). Another novel approach is inhibiting telomerase activity leading to disrupting cacner cell immortality with imetelstat, perifosine, KML001, and BIBR1532, or with immunotherapeutic peptides targeting the catalytic subunit hTERT, including GV1001, P540, GX301, and Vx-001 (Niu et al., 2021; Wang and Deng, 2023).

### 4.3 Immunotherapies

HCC responds to immunotherapies, mainly immune checkpoint inhibitors (ICIs), which demonstrated superior effectiveness to sorafenib alone when combined with anti-angiogenic agents (Niu et al., 2021). This was confirmed by IMbrave150 (atezolizumab and bevacizumab), HIMALAYA/STRIDE (durvalumab and tremelimumab), and rivoceranib and camrelizumab have significantly improved OS compared to sorafenib. ICIs can complement or follow locoregional treatments to eliminate residual disease (Li et al., 2024). Eight trials comprising 6,290 patients were integrated into a meta-analysis to find that the atezolizumab/bevacizumab combination significantly outperformed sorafenib (HR 0.58), nivolumab (HR 0.68), and lenvatinib (HR 0.63) in terms of OS (Wang and Deng, 2023). Therefore, this combination is the current preferred first-line therapy for unresectable or advanced HCC (medscape, 2024). Despite these advancements, challenges remain due to modest survival benefits, high treatment costs, and substantial toxicity, highlighting a persistent need for more effective and accessible therapies.

## 5 Sodium homeostasis: a novel target for HCC therapy?

Sodium homeostasis is an under-researched field in cancer. Several indirect and direct evidence, however, associate [Na+]i variations with crucial features of transformed tissue and suggest that disruption of Na + homeostasis might be a potential molecular mechanism for therapeutic interventions for HCC and cancer in general.

### 5.1 Na+ and reverse pH gradient in cancer

In cancer, the intracellular alkalosis and the acidic extracellular environment create a reverse pH gradient compared with untransformed tissue. This reverse pH gradient favors cancer cell division, migration and resistance to chemotherapy and hypoxia. pH-regulating proteins are, thus, receiving growing scientific attention as oncologic targets. Their critical importance in untransformed tissue, however, makes it difficult to safely manipulate their functionality.

Intracellular alkalosis occurs as a paradoxical reaction to the increase of acidic molecules produced by the glycolytic ATP production (Warburg effect) and it is due to the augmented expression of Na+-independent and Na+-dependent pH controlling systems (i.e., Na+/H+ and Na+-HCO3- exchanger) (Webb et al., 2011; Parks et al., 2013). Together with Na+-dependent pH regulatory proteins, Na + transporters are generally upregulated in HCC and cancer in general (i.e., Na+/Ca2+ exchanger, Na+/K+/2Cl- and Na+/glucose co-transporters) or are expressed in HCC but not in the corresponding un-transformed tissue (acid sensitive ion channels). They all operate favoring a net Na + influx from the extracellular space into the cytosol, thus elevating [Na+]i. (Leslie et al., 2019). Significantly, this phenomenon was recently demonstrated in human cancers. Non-invasive 23Na-magnetic resonance imaging analysis of human malignant gliomas, breast and prostate tumors confirmed a higher concentration of Na+i and Na+e compared to surrounding normal tissues (Ouwerkerk et al., 2003; Jacobs et al., 2004; Barrett et al., 2018; Ruggiero et al., 2018; Mohamed et al., 2021). Studies employing “*in vivo* Field-Cycling Relaxometry”, parallelly, evidenced an augmented water exchange in human cancer tissues, indicating an increased intracellular osmotic pressure in tumors that correlated to cancer aggressiveness (28). These observations showed that the reverse pH gradient is associated with the tendency of cancer cells to accumulate and maintain high levels of intracellular sodium.

### 5.2 Na+ and death and survival of primary hepatocytes

Early studies explored the role of [Na+]i variations in death and resistance to death of un-transformed rodent hepatocytes. They demonstrated that Na + overload preceded primary hepatocyte death induced by various toxic conditions affecting mitochondrial activity and energy production, and that preventing Na + increase delayed the appearance of cell death. They also showed that the irreversible increase of intracellular Na+ was associated to volume deregulation and to the appearance of cell death following ATP depletion (Carini et al., 1995; Carini et al., 1999). Subsequent investigations on the intrinsic protective mechanisms of hepatic preconditioning additionally demonstrated that the increased resistance to hypoxic damage of preconditioned hepatocytes was causally associated with the maintenance of Na + homeostasis (Carini et al., 2000; Alchera et al., 2008).

### 5.3 Anticancer activity of the sodium ionophore Monensin

Monensin is a sodium and proton selective ionophore that facilitates sodium ion entrance into the cells (PubChem, 2025). It works by forming complexes with sodium ions (Na^+^), which pass through the cell membrane due to the high lipophilicity, then release the sodium ion in the cytoplasm (Figures 1B,C). It is classified as a coccidiostat and nonhormonal growth promoter in veterinary medicine (Aowicki and Huczyński, 2013). The activity of Monensin against several tumors has been demonstrated in many studies on cancer cells and animal models. These studies include HCC (Clemente et al., 2023), breast cancer (Fiorilla et al., 2025), ovarian cancer (Yao et al., 2021), neuroblastoma (Serter Kocoglu et al., 2023), chemo-resistant pancreatic cancer (Wang et al., 2018), prostate cancer (Barrett et al., 2018), ovarian and pancreatic tumors and colorectal cancer (Zhou et al., 2023).

Previously, the anticancer effect of Monensin was thought to be based on cytostatic properties, modifying intracellular signaling such as Wnt/β-catenin and/or growth factor-activated signal pathways (particularly, EGFR) (Deng et al., 2015; Wang et al., 2018).

However, novel observations appeared to rule out this theory, showing that the cytostatic action of Monensin described in the *in vitro* studies depended by the cell colture settings and was not detectable *in vivo* (Fiorilla et al., 2025). In particular Monensin demonstrated to reduce triple-negative breast cancer (TNBC) development without displaying anti-proliferative effect on malignant tissues, evident by no significant changes in Proliferating Cell Nuclear Antigen (PCNA) and Ki-67 expression (Fiorilla et al., 2025). Moreover, in previous experiments on HCC allograft, Monensin showed to significantly reduce tumor growth without changing Ki-67 levels in the tumors, bone marrow, or intestines and without affecting hemopoiesis (Clemente et al., 2023).

Monensin’s activity resulted instead related to a tumor-selective sodium-dependent cytotoxicity, which was not associated to the alteration of the integrity of normal tissues. Monensin caused in fact intracellular sodium overload and ATP depletion in cancer cells, leading to expansion of the necrotic area in the tumor mass (Clemente et al., 2023; Fiorilla et al., 2025).

### 5.4 Monensin inhibits HCC by inducing a specific and sodium-dependent HCC cells killing

In the HCC allograft model, Monensin selectively and furtherly increased intracellular sodium in mice hepatocarcima cells and in HCC mice allograft but not in primary hepatocytes and in healthy tissues. This cancer-specific sodium increase was directly related “*in vitro*” with energy depletion, increase of mitochondrial sodium content and decrease of basal and ATP- linked mitochondrial O_2_ consumption, enhancement of intracellular water life-time and killing of mice hepatoma cells and “*in vivo*” with the regression of allograft tumors in immunodeficient mice, with extensive necrosis of tumors and no effect on healthy tissues (Clemente et al., 2023). These findings suggest that in HCC, the already high intracellular sodium concentration and the continuous sodium influx consequent to the glycolytic metabolism, make the cancer cells unable to energetically cope to the additional sodium influx induced by Monensin with irreversible sodium overload. Such condition initiates a death cascade involving mitochondrial impairment and ATP depletion that ultimately leads to an uncontrolled osmotic swelling and cell death (Figures 1D,E). The tumor-specific action of Monensin, along with its non-measurable toxicity in healthy tissues, highlights its promise as a therapeutic agent for liver cancer.

### 5.5 Challenges of translating the disruption of cancer sodium homeostasis for HCC therapy

Currently, there are no approved therapies based on sodium homeostasis de-regulations for the treatment of NASH or HCC and cancer in general.

Clinical studies have tested the efficacy of Na^+^/K^+^ ATPase inhibitors alone or in combination with other anticancer drugs in cancer patients. Na^+^/K^+^ ATPase is upregulated in cancer, and its inhibition has potent anticancer effects in pre-clinical models (Durlacher et al., 2015). Na^+^/K^+^ ATPase ubiquity and its fundamental role for cell volume and cation gradient maintenance, however, argues against the safety of its generalized employment in therapy.

Epithelial Sodium Channel (ENaC), and ASIC (acid-sensing ion channel) are constitutively active and proton-gated Na + channels that transfer Na^+^ ions into the cytoplasm from the extracellular spaces (Hanukoglu, 2017). Both EnaC and ASIC are overexpressed in HCC and other extrahepatic cancers (Wang et al., 2022) but, in normal tissues, EnaC are widely represented in several organs while ASIC are mainly confined to nervous systems (Gründer et al., 2024). The restricted localization of ASIC and the possible repositioning of the ASIC inhibitor drugs clinically used for epilepsy and psychiatric diseases make ASIC particularly attractive onco-targets (Wang et al., 2022). However, ASIC inhibitors appear effective in preventing EMT and cancer or HCC cells migration by altering the acidic extracellular pH (Jin et al., 2015) but are not likely to massively affect intracellular sodium concentration and are not reported to inhibit cancer growth. Moreover, these drugs are prone to causing QT interval prolongation, and NASH patients often have comorbid cardiovascular diseases, which could be a significant obstacle to clinical application.

Sodium ionophores like Monensin demonstrated potent anticancer effects in animals models but they have been not yet tested in clinical studies and Monensin safety studies in humans are limited. A case report (Blain et al., 2017) described 8 days of abdominal pain combined with vomiting in a 58-year-old man who orally took 300 mg (4.6 mg/kg) of Monensin. The patient suffered from increased rhabdomyolysis and creatinine kinase, but the effects were resolved 2 months following his discharge. However, the patient already had toxoplasmosis, which could have affected his system before the Monensin exposure. Another report included two patients, one of whom had irreversible cardiopulmonary disease, while the other recovered (Zhang et al., 2021). The toxic dose of Monensin varies based on species, with 2–3, 26, and 200 mg/kg considered lethal for horses, cattle, and chickens, respectively (Todd et al., 1984). Yet no clear data is available about the usage of Monensin in therapeutic doses in humans, or a detailed description of the clinical manifestations upon direct administration under medical monitoring.

## 6 Discussion

This study summarizes the available therapeutic approaches for HCC and highlights the potentiality of exploring the higher basal sodium concentration in cancer cells, with sodium ionophores, such as Monensin as a novel targetable biomarker. Monensin is showing significant activity against several tumors, many of which lack effective therapies and have a poor prognosis, such as HCC. Its precise molecular mechanism of action, toxicity in humans, and effectiveness in translationally relevant animal models are underexplored. The intracellular alkaline pH of cancer cells is a paradoxical response to the acidic species produced by the Warburg effect and involves the activation or increased expression of cation transporters, including sodium transporters. Thus, the observed increase in intracellular sodium of cancer tissue is proposed to be a correlated consequence.

Available *in vivo* evidence indicates that Monensin has negligible adverse effects in mice and no cytostatic properties. These supports conducting deeper research in an interdisciplinary approach to better characterize Monensin molecular effects, employing drug discovery tools to develop novel molecules, and identifying further clinical biomarkers indicative of sodium ionophore sensitivity, aligned with precision medicine principles. Besides leading to plasma membrane mechanical lysis, the disruption of sodium homeostasis may affect intracellular calcium concentrations. The latter might produce additional anti-cancer effects, such as modulating autophagy, lipogenesis, or activating immune cells. Future research devoted to such still unexplored aspects of Monensin activity might enlighten novel therapeutic properties of sodium ionophores that can either potentiate their cancer-selective cytotoxic action or compensate possible rising resistant genotypes of HCC cells to cell death by sodium and water overload.

## References

[B1] Alchera E. Tacchini L. Imarisio C. Dal Ponte C. De Ponti C. Gammella E. (2008). Adenosine-dependent activation of hypoxia-inducible factor-1 induces late preconditioning in liver cells. Hepatology 48, 230–239. 10.1002/hep.22249 18506850

[B2] Aowicki D. Huczyński A. (2013). Structure and antimicrobial properties of monensin A and its derivatives: summary of the achievements. Biomed. Res. Int. 2013, 742149. 10.1155/2013/742149 23509771 PMC3586448

[B3] Ayuso C. Rimola J. Vilana R. Burrel M. Darnell A. García-Criado Á. (2018). Diagnosis and staging of hepatocellular carcinoma (HCC): current guidelines. Eur. J. Radiol. 101, 72–81. 10.1016/j.ejrad.2018.01.025 29571804

[B4] Barrett T. Riemer F. McLean M. A. Kaggie J. Robb F. Tropp J. S. (2018). Quantification of total and intracellular sodium concentration in primary prostate cancer and adjacent normal prostate tissue with magnetic resonance imaging. Invest. Radiol. 53, 450–456. 10.1097/RLI.0000000000000470 29969108

[B6] Bialecki E. S. Di Bisceglie A. M. (2005). Diagnosis of hepatocellular carcinoma. HPB 7, 26–34. 10.1080/13651820410024049 18333158 PMC2023919

[B7] Blain M. Garrard A. Poppenga R. Chen B. Valento M. Halliday Gittinger M. (2017). Survival after severe rhabdomyolysis following monensin ingestion. J. Med. Toxicol. 13, 259–262. 10.1007/s13181-017-0616-6 28516409 PMC5570726

[B8] Carini R. Bellomo G. Benedetti A. Fulceri R. Gamberucci A. Parola M. (1995). Alteration of Na+ homeostasis as a critical step in the development of irreversible hepatocyte injury after adenosine triphosphate depletion. Hepatology 21, 1089–1098. 10.1002/hep.1840210431 7705784

[B9] Carini R. Autelli R. Bellomo G. Albano E. (1999). Alterations of cell volume regulation in the development of hepatocyte necrosis. Exp. Cell Res. 248, 280–293. 10.1006/excr.1999.4408 10094834

[B10] Carini R. De Cesaris M. G. Splendore R. Bagnati M. Albano E. (2000). Ischemic preconditioning reduces Na(+) accumulation and cell killing in isolated rat hepatocytes exposed to hypoxia. Hepatology 31, 166–172. 10.1002/hep.510310125 10613742

[B11] Cheng A.-L. Kang Y.-K. Chen Z. Tsao C.-J. Qin S. Kim J. S. (2009). Efficacy and safety of sorafenib in patients in the Asia-Pacific region with advanced hepatocellular carcinoma: a phase III randomised, double-blind, placebo-controlled trial. Lancet Oncol. 10, 25–34. 10.1016/S1470-2045(08)70285-7 19095497

[B13] Clemente N. Baroni S. Fiorilla S. Tasso F. Reano S. Borsotti C. (2023). Boosting intracellular sodium selectively kills hepatocarcinoma cells and induces hepatocellular carcinoma tumor shrinkage in mice. Commun. Biol. 6, 574. 10.1038/s42003-023-04946-4 37248274 PMC10227045

[B14] Deng Y. Zhang J. Wang Z. Yan Z. Qiao M. Ye J. (2015). Antibiotic monensin synergizes with EGFR inhibitors and oxaliplatin to suppress the proliferation of human ovarian cancer cells. Sci. Rep. 5, 17523. 10.1038/srep17523 26639992 PMC4671000

[B17] Durlacher C. T. Chow K. Chen X. W. He Z. X. Zhang X. Yang T. (2015). Targeting Na^+^/K^+^ -translocating adenosine triphosphatase in cancer treatment. Clin. Exp. Pharmacol. Physiol. 42, 427–443. 10.1111/1440-1681.12385 25739707

[B18] Fiorilla S. Tasso F. Clemente N. Trisciuoglio T. Boldorini R. Carini R. (2025). Monensin inhibits triple-negative breast cancer in mice by a Na+-dependent cytotoxic action unrelated to cytostatic effects. Cells 14, 185. 10.3390/cells14030185 39936977 PMC11817484

[B20] Gründer S. Vanek J. Pissas K. P. (2024). Acid-sensing ion channels and downstream signalling in cancer cells: is there a mechanistic link? Pflugers Arch. 476, 659–672. 10.1007/s00424-023-02902-z 38175291 PMC11006730

[B22] Hanukoglu I. (2017). ASIC and ENaC type sodium channels: conformational states and the structures of the ion selectivity filters. FEBS J. 284, 525–545. 10.1111/febs.13840 27580245

[B23] Huang D. Q. El-Serag H. B. Loomba R. (2021). Global epidemiology of NAFLD-related HCC: trends, predictions, risk factors and prevention. Nat. Rev. Gastroenterol. Hepatol. 18, 223–238. 10.1038/s41575-020-00381-6 33349658 PMC8016738

[B24] Jacobs M. A. Ouwerkerk R. Wolff A. C. Stearns V. Bottomley P. A. Barker P. B. (2004). Multiparametric and multinuclear magnetic resonance imaging of human breast cancer: current applications. Technol. Cancer Res. Treat. 3, 543–550. 10.1177/153303460400300603 15560711

[B26] Jin C. Ye Q. H. Yuan F. L. Gu Y. L. Li J. P. Shi Y. H. (2015). Involvement of acid-sensing ion channel 1α in hepatic carcinoma cell migration and invasion. Tumour Biol. 36, 4309–4317. 10.1007/s13277-015-3070-6 25613068

[B27] Koshy A. (2025). Evolving global etiology of hepatocellular carcinoma (HCC): insights and trends for 2024. J. Clin. Exp. Hepatol. 15, 102406. 10.1016/j.jceh.2024.102406 39346785 PMC11426038

[B29] Leslie T. K. James A. D. Zaccagna F. Grist J. T. Deen S. Kennerley A. (2019). Sodium homeostasis in the tumour microenvironment. Biochim. Biophys. Acta Rev. Cancer 1872, 188304. 10.1016/j.bbcan.2019.07.001 31348974 PMC7115894

[B30] Li M. Bhoori S. Mehta N. Mazzaferro V. (2024). Immunotherapy for hepatocellular carcinoma: the next evolution in expanding access to liver transplantation. J. Hepatol. 81, 743–755. 10.1016/j.jhep.2024.05.037 38848767

[B31] Llovet J. M. Ricci S. Mazzaferro V. Hilgard P. Gane E. Blanc J.-F. (2008). Sorafenib in advanced hepatocellular carcinoma. N. Engl. J. Med. 359, 378–390. 10.1056/NEJMoa0708857 18650514

[B32] Makary M. S. Khandpur U. Cloyd J. M. Mumtaz K. Dowell J. D. (2020). Locoregional therapy approaches for hepatocellular carcinoma: recent advances and management strategies. Cancers 12, 1914. 10.3390/cancers12071914 32679897 PMC7409274

[B33] Medscape (2024). Hepatocellular carcinoma (HCC): practice essentials, anatomy, pathophysiology. Available online at: https://emedicine.medscape.com/article/197319-overview (Accessed November 30, 2024).

[B34] Mohamed S. Adlung A. Ruder A. M. Hoesl M. A. U. Schad L. Groden C. (2021). MRI detection of changes in tissue sodium concentration in brain metastases after stereotactic radiosurgery: a feasibility study. J. Neuroimaging Off. J. Am. Soc. Neuroimaging 31, 297–305. 10.1111/jon.12823 33351997

[B35] Niu M. Yi M. Li N. Wu K. Wu K. (2021). Advances of targeted therapy for hepatocellular carcinoma. Front. Oncol. 11, 719896. 10.3389/fonc.2021.719896 34381735 PMC8350567

[B36] Ouwerkerk R. Bleich K. B. Gillen J. S. Pomper M. G. Bottomley P. A. (2003). Tissue sodium concentration in human brain tumors as measured with 23Na MR imaging. Radiology 227, 529–537. 10.1148/radiol.2272020483 12663825

[B37] Parks S. K. Chiche J. Pouysségur J. (2013). Disrupting proton dynamics and energy metabolism for cancer therapy. Nat. Rev. Cancer 13, 611–623. 10.1038/nrc3579 23969692

[B38] PubChem (2025). Monensin sodium. Available online at: https://pubchem.ncbi.nlm.nih.gov/compound/23690927.

[B39] Reig M. Forner A. Rimola J. Ferrer-Fàbrega J. Burrel M. Garcia-Criado Á. (2022). BCLC strategy for prognosis prediction and treatment recommendation: the 2022 update. J. Hepatol. 76, 681–693. 10.1016/j.jhep.2021.11.018 34801630 PMC8866082

[B40] Roncalli M. (2009). Pathologic diagnosis of early hepatocellular carcinoma: a report of the international consensus group for hepatocellular neoplasia. Available online at: https://air.unimi.it/handle/2434/49917 (Accessed November 30, 2024). 10.1002/hep.22709 19177576

[B41] Ruggiero M. R. Baroni S. Pezzana S. Ferrante G. Geninatti Crich S. Aime S. (2018). Evidence for the role of intracellular water lifetime as a tumour biomarker obtained by *in vivo* field-cycling relaxometry. Angew. Chem. Int. Ed. Engl. 57, 7468–7472. 10.1002/anie.201713318 29575414 PMC6175164

[B42] Rumgay H. Arnold M. Ferlay J. Lesi O. Cabasag C. J. Vignat J. (2022). Global burden of primary liver cancer in 2020 and predictions to 2040. J. Hepatol. 77, 1598–1606. 10.1016/j.jhep.2022.08.021 36208844 PMC9670241

[B44] Serter Kocoglu S. Oy C. Secme M. Sunay F. B. (2023). Investigation of the anticancer mechanism of monensin via apoptosis-related factors in SH-SY5Y neuroblastoma cells. Clin. Transl. Sci. 16, 1725–1735. 10.1111/cts.13593 37477356 PMC10499413

[B45] Taiho Pharmaceutical Co., Ltd (2019). A randomized, double-blind, placebo-controlled phase III trial of TSU-68 in combination with transcatheter arterial chemoembolization in patients with unresectable hepatocellular carcinoma. Clinicaltrials.gov. Available online at: https://clinicaltrials.gov/study/NCT01465464 (Accessed July 19, 2025).

[B46] Todd G. C. Novilla M. N. Howard L. C. (1984). Comparative toxicology of monensin sodium in laboratory animals. J. Anim. Sci. 58, 1512–1517. 10.2527/jas1984.5861512x 6746442

[B47] Wang X. Wu X. Zhang Z. Ma C. Wu T. Tang S. (2018). Monensin inhibits cell proliferation and tumor growth of chemo-resistant pancreatic cancer cells by targeting the EGFR signaling pathway. Sci. Rep. 8, 17914. 10.1038/s41598-018-36214-5 30559409 PMC6297164

[B57] Wang Y. Deng B. (2023). Hepatocellular carcinoma: molecular mechanism, targeted therapy, and biomarkers. Cancer Metastasis Rev. 42, 629–652. 10.1007/s10555-023-10084-4 36729264

[B48] Wang Y. Zhou H. Sun Y. Huang Y. (2022). Acid-sensing ion channel 1: potential therapeutic target for tumor. Biomed. Pharmacother. 155, 113835. 10.1016/j.biopha.2022.113835 36271585

[B49] Webb B. A. Chimenti M. Jacobson M. P. Barber D. L. (2011). Dysregulated pH: a perfect storm for cancer progression. Nat. Rev. Cancer 11, 671–677. 10.1038/nrc3110 21833026

[B50] Wegermann K. Hyun J. Diehl A. M. (2021). Molecular mechanisms linking nonalcoholic steatohepatitis to cancer. Clin. Liver Dis. 17, 6–10. 10.1002/cld.1006 33552478 PMC7849296

[B51] Yao S. Wang W. Zhou B. Cui X. Yang H. Zhang S. (2021). Monensin suppresses cell proliferation and invasion in ovarian cancer by enhancing MEK1 SUMOylation. Exp. Ther. Med. 22, 1390–10. 10.3892/etm.2021.10826 34650638 PMC8506924

[B53] Zhang Z. Cui S. Zhang J. (2021). Rhabdomyolysis and hepatotoxicity following accidental monensin ingestion: a report of two cases. Toxicol. Ind. Health 37, 34–37. 10.1177/0748233720974128 33305694

[B54] Zhang T. Nie Y. Wang J. (2023). The emerging significance of mitochondrial targeted strategies in NAFLD treatment. Life Sci. 329, 121943. 10.1016/j.lfs.2023.121943 37454757

[B55] Zhou Y. Deng Y. Wang J. Yan Z. Wei Q. Ye J. (2023). Effect of antibiotic monensin on cell proliferation and IGF1R signaling pathway in human colorectal cancer cells. Ann. Med. 55, 954–964. 10.1080/07853890.2023.2166980 36896461 PMC10795625

[B56] Zhu A. X. Kang Y.-K. Yen C.-J. Finn R. S. Galle P. R. Llovet J. M. (2019). Ramucirumab after sorafenib in patients with advanced hepatocellular carcinoma and increased α-fetoprotein concentrations (REACH-2): a randomised, double-blind, placebo-controlled, phase 3 trial. Lancet Oncol. 20, 282–296. 10.1016/S1470-2045(18)30937-9 30665869

